# Epigenetic Regulation of the Biosynthesis & Enzymatic Modification of Heparan Sulfate Proteoglycans: Implications for Tumorigenesis and Cancer Biomarkers

**DOI:** 10.3390/ijms18071361

**Published:** 2017-06-26

**Authors:** Elizabeth E. Hull, McKale R. Montgomery, Kathryn J. Leyva

**Affiliations:** 1 Biomedical Sciences Program, College of Health Sciences, Midwestern University, Glendale, AZ 85308, USA; mmontg@midwestern.edu; 2 Department of Microbiology & Immunology, Arizona College of Osteopathic Medicine, Midwestern University, Glendale, AZ 85308, USA; kleyva@midwestern.edu

**Keywords:** biomarkers, biosynthetic pathways, enzymatic modification, epigenetic regulation, glycosylation, heparan sulfate proteoglycans, lectin arrays, sulfation

## Abstract

Emerging evidence suggests that the enzymes in the biosynthetic pathway for the synthesis of heparan sulfate moieties of heparan sulfate proteoglycans (HSPGs) are epigenetically regulated at many levels. As the exact composition of the heparan sulfate portion of the resulting HSPG molecules is critical to the broad spectrum of biological processes involved in oncogenesis, the epigenetic regulation of heparan sulfate biosynthesis has far-reaching effects on many cellular activities related to cancer progression. Given the current focus on developing new anti-cancer therapeutics focused on epigenetic targets, it is important to understand the effects that these emerging therapeutics may have on the synthesis of HSPGs as alterations in HSPG composition may have profound and unanticipated effects. As an introduction, this review will briefly summarize the variety of important roles which HSPGs play in a wide-spectrum of cancer-related cellular and physiological functions and then describe the biosynthesis of the heparan sulfate chains of HSPGs, including how alterations observed in cancer cells serve as potential biomarkers. This review will then focus on detailing the multiple levels of epigenetic regulation of the enzymes in the heparan sulfate synthesis pathway with a particular focus on regulation by miRNA and effects of epigenetic therapies on HSPGs. We will also explore the use of lectins to detect differences in heparan sulfate composition and preview their potential diagnostic and prognostic use in the clinic.

## 1. Introduction

### 1.1. Structure and Types of Heparan Sulfate Proteoglycans (HSPGs)

Heparan sulfate proteoglycans (HSPGs) are a diverse group of glycoproteins composed of one or more chains of heparan sulfate (HS) covalently bound to a core protein through a tetrasaccharide bridge. HSPGs vary considerably in molecular mass, from ~10 to over 400 kDa (as reviewed in [1]) depending on the nature of the core protein as well as the number and length of HS chains. There is limited diversity in the protein core structure [2], with most variability observed within the HS chains. More than 25 enzymes have been identified that are involved in HS synthesis and modification [3]. Due to their sulfation, the HS chains are negatively charged and can bind to many different ligands at the cell surface, within the extracellular matrix, and within the plasma [4]. HSPGs can be broadly classified into three main categories depending on their cellular/tissue location: (1) plasma membrane-associated; (2) secreted into the extracellular matrix; and (3) within secretory vesicles (as reviewed in [1]).

The major plasma membrane-associated HSPGs are the syndecans and the glypicans [1,4,5]. Structurally, the syndecans (syndecan-1, -2, -3, and -4) are transmembrane proteins composed of an N-terminal signal sequence, an ectodomain, a hydrophobic transmembrane domain, and a short C-terminal cytosolic tail. The addition of the HS chain occurs post-translationally on a serine residue. In contrast to syndecans, glypicans are not transmembrane glycoproteins but are extracellularly attached to the membrane via a glycosylphosphatidylinositol (GPI) anchor. There are six glypican family members (GPC1–6) that can be further classified into two subfamilies: GPC1/2/4/6 and GPC3/5 based on sequence similarity. Structurally, glypicans contain a hydrophobic domain at the C-terminus, allowing for the attachment of the protein to the GPI anchor. Unlike the syndecans, the HS chains on glypicans are covalently bound near the C-terminus, resulting in chains which are very close to the cell surface and can be liberated from the cell by lipase activity [6,7]. Most of the GPI-anchored glypicans are found within lipid rafts on the apical side of the cell [8,9,10], but this is not exclusive. Minor membrane-associated HSPGs, also known as “part-time HSPGs”, include betaglycan, neuropilin-1, and CD44v3 and are single-pass transmembrane receptors. Betaglycan, also known as transforming growth factor β receptor III (TGFβR3), contains either heparan sulfate (HS) or chondroitin sulfate (CS) chains and functions as a co-receptor for the TGFβ superfamily [11,12]. Neuropilin-1 also contains either HS or CS chains and functions as a mediator of angiogenesis and axonal guidance by regulating cellular responses to vascular endothelial growth factor (VEGF) [13,14,15] and semaphorins [16]. CD44v3, a splice variant of CD44, is an HS-containing transmembrane receptor for hyaluronan and has been shown to promote tumor growth and metastasis in breast cancer [17] and head and neck squamous cell carcinoma [18,19].

The second category of HSPGs are those secreted into the extracellular matrix (ECM). These include perlecan, agrin, and collagen XVIII, which are all large, multidomain proteins (reviewed in [1,20,21]). Perlecan, synthesized and secreted by endothelial and vascular smooth muscle cells, binds and cross-links various ECM and cell-membrane components. The HS chains are bound to perlecan at the N-terminal domain. Binding of HS chains to perlecan is not required for proper protein folding or secretion, but decreases in number and/or sulfation has been shown to reduce perlecan function. Agrin is secreted by neuronal cells into the ECM and is important in the formation of the neuromuscular junction during embryogenesis. Agrin contains up to three heparan sulfate attachment sites, although most studies have shown that only two HS chains are typically found on the secreted form. Collagen XVIII, a ubiquitously expressed component of basement membranes, has a C-terminal domain known as endostatin that functions to inhibit angiogenesis and tumor growth when cleaved.

The last category of HSPGs are those found within secretory vesicles. At this time, the only characterized proteoglycan within this category is serglycin. Serglycin is primarily expressed in hematopoietic and endothelial cells and serves an important role in formation and retention of inflammatory mediators inside storage granules and secretory vesicles [1,22].

### 1.2. General Functions of HSPGs

A major determinant of HSPG function is the pattern of glycosylation and sulfation of the HS chains, which is highly controlled by the cells/tissues expressing the HSPG [3]. The ligands that HSPGs bind are also quite varied and include, but are not limited to, cell surface receptors, extracellular matrix proteins, growth factors, cytokines and morphogens (reviewed in [21]).

**Figure 1:**
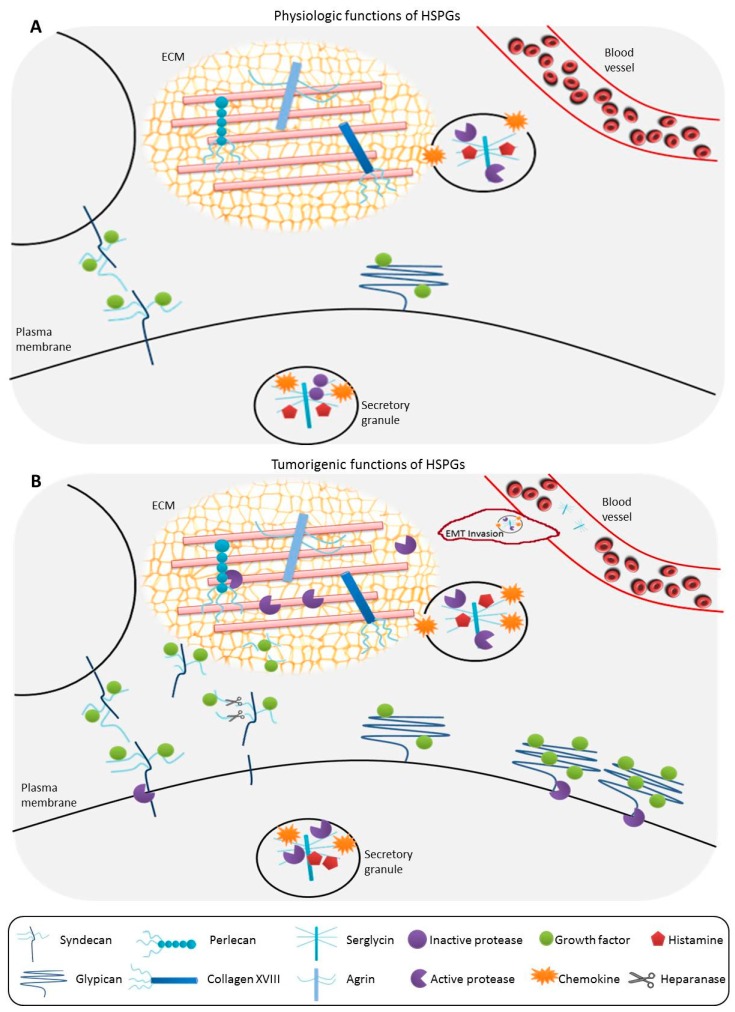
(**A**) Under physiologic conditions, syndecans are located at the cell surface, functioning as growth factor receptors and are important for cell-to-cell communication. Glypicans are also located at the cell surface, attached to the membrane through a glycosylphosphatidylinositol (GPI) anchor, and function as growth factor receptors. Perlecan, agrin, and serglycin are found within the extracellular matrix (ECM) and aid in the formation and structural integrity of the ECM barrier. Serglycins are the only intracellular heparan sulfate proteoglycans (HSPGs), found in secretory granules with chemokines and histamine, and function in maintaining proteases in their inactive form. Upon secretion, serglycins activate ECM proteases and are important in regulation of host defenses and wound repair; (**B**) In tumorigenesis, syndecans can be proteolytically cleaved, and these soluble syndecans can sequester growth factors in the ECM. Heparanases can cleave HS chains, which can also bind and complex with growth factors in the ECM. Glypican expression at the cell surface is often upregulated, resulting in increased growth factor binding and uptake which mediates tumor cell growth. Tumor cells have increased serglycin secretion, causing enhanced protease activity, facilitating ECM breakdown that promotes tumor invasiveness and metastasis.

Due to the large diversity in HSPGs and their ligands, it is naturally unsurprising that the collective functions ascribed to HSPGs are just as numerous (as reviewed in [1,23,24]). HSPGs are involved in the development of the basement membrane barrier, providing a framework for epithelial support, regulating transport of solutes, and promoting the extravasation of cells during inflammatory responses [23,25,26,27]. HSPGs that are located at the cell surface are also involved in the establishment of morphogen and chemokine gradients important in WBC extravasation, but are also vital during development [28,29,30]. HSPGs located within secretory vesicles are involved in the packaging of vesicular contents, maintenance of protease activity, and regulating various activities upon secretion, such as host defense mechanisms and wound repair (e.g., [22,31,32,33]). Membrane-associated HSPGs are also involved as receptors or coreceptors on the cell surface promoting a variety of activities: (1) they can function as coreceptors for growth factor receptors, mediating signal transduction pathways (e.g., [34,35,36]); (2) they can function as endocytic receptors, facilitating the transcytosis and/or clearance of lipoproteins [37,38] and promoting exosome uptake [39,40]; (3) they can cooperate with cell adhesion molecules, such as integrins, to affect cellular migration (e.g., [27,41,42]); (4) they can bind to and regulate the activity of matrix metalloproteases within the ECM (e.g., [43]); and (5) they can act to mediate cytokine-induced signal transduction pathways (e.g., [44,45]) (Figure 1A). Given how many essential cellular and developmental processes involve the activity of HSPGs, it is not surprising that modifications or alterations in HSPG structure could impart a dysregulation in function and potentially lead to the development of cancer (Figure 1B).

### 1.3. Alterations of HSPGs Serving as Biomarkers in Cancer

Several HSPGs have been shown to be upregulated in many cancers and can serve as biomarkers for cancer diagnosis and/or prognosis. As one example, neuropilin-1 has been shown to be a prognostic indicator for tumor metastasis in oral squamous cell carcinoma [46], prostate cancer [47], and malignant melanoma [48]. However, the remodeling of HSPGs through enzymatic modification of HS chains is associated with malignant transformation of cells, and can potentially serve as molecular biomarkers to aid in the diagnosis and prognosis of cancer. Alterations in glycosylation of HSPGs can facilitate the metastasis of cancer cells by affecting cellular adhesion. As one example, Ferguson and Datta reported that activity of heparan sulfate 2-*O*-sulfotransferase (2OST) was critical in invasion of LNCaP-C4-2B prostate cancer cells [49]. In their study, they documented increases in E-cadherin and actin expression at the cell surface upon 2OST knockdown, indicating stable adherens junction formation. Additionally, activity of 2OST was increased in response to stress-inducible transcription factors, which these authors hypothesize would be elevated in cancer progression.

One of the first reports indicating that structural alterations in glycosylation can result in tumorigenesis is in hereditary multiple exostosis and chondrosarcoma [50]. Most of these cases arise due to mutations in the exostosin (EXT) genes *EXT1* and *EXT2*, encoding enzymes involved in biosynthesis of heparan sulfate [51], and further work demonstrated that these mutations interfere with proper glycosylation and function of HSPGs [52]. Since these early reports, numerous studies have been published that provide evidence of structural alterations of HSPGs and development of cancer. In a recent study by Jao et al. [53] using a mouse knockout model, they showed that loss of *N*-deacetylase and *N*-sulfotransferase 4 (NDST4), an enzyme involved in HS sulfation, may result in tumorigenesis and progression of colorectal cancer. As reviewed in [54], a loss of syndecan-1 is associated with a decrease in E-cadherin expression, which can alter the adhesion and migration properties in some tumors; this loss of syndecan-1 is associated with accelerated tumor progression and poor prognosis in head and neck, lung, and colorectal cancer. Glypican-3 (GPC3) is well characterized as a negative regulator of cell growth and functions as a tumor suppressor protein. Several studies have shown that aberrant expression of GPC3 in hepatocellular carcinoma [55,56] and urothelial cancer [57] can serve as a biomarker for diagnosis and prognosis. Wade et al. [58] reported that alterations in activity of the sulfatase enzymes SULF1 and SULF2 promote receptor tyrosine kinase signaling and progression in glioblastoma, and suggest that these alterations “are promising biomarkers for disease and therapeutic targets.” Not only can alterations occur between cancerous and non-cancerous cells, Fernandez-Vega et al. [59] reported that HS modifications may also be different in right-sided colorectal cancer (CRC), depending on the stage of the tumor. They reported increased expression of five genes encoding HSPG-modifying enzymes (See Table 1) between normal and non-metastatic CRC tissue, but when comparing normal tissues with metastatic CRC tissue, these same five genes were not differentially expressed. In total, less than 20% of the genes involved in HSPG biosynthesis were differentially expressed in metastatic tumors versus nearly 40% in non-metastatic tumors [59], allowing for the potential of these genes to serve as biomarkers for staging of colorectal cancer.

Based on the compilation of reports documenting HSPGs and their influence in tumorigenesis, it is now accepted that structural alterations in the HS chains, typically assessed using antibody-based techniques, can serve as biomarkers for various cancers. Normal HS biosynthesis is variable and highly regulated within tissues, allowing for coordinated cellular responses to ligand binding; these processes can be commandeered by cancer cells, resulting in changes to the structure, degree of expression, and/or function of HSPGs within cancer cells [4]. As Kreuger and Kjeller [60] point out: “The key to understanding the function of HSPGs [and their utility as biomarkers] is to clarify how HS biosynthesis is regulated in different biological contexts.” The following sections will address the regulation of HS biosynthesis in the context of cancer.

## 2. Heparan Sulfate Biosynthetic Pathway

### 2.1. Synthesis of the Serine-Linked Tetrasaccharide Linker

All HS moieties are linked to a serine residue on the core protein by a tetrasaccharide (Xyl-Gal-Gal-GlcA). The initial and apparently rate limiting step in HS chain synthesis is the addition of xylose (catalyzed by Xylt1/2 or XYLT1/2) to a serine on the core protein [61,62]. Little is known about the subsequent three enzymes in the synthesis of this tetrasaccharide linkage. Most recent reviews present these enzymes as Galt1/2 and Glcat1 but these names are non-standard. The formation of the β-4 xyl-gal linkage (described as Galt1) can be catalyzed by a family of seven β-4 galactosyl transferases (B4GALT) which transfer galactose to a variety of sugars including xylose but B4GALT7 appears to predominate [63]. The formation of the β-3 gal-gal linkage (described as Galt2) is more appropriately known as B3GALT6 while the enzyme catalyzing the β-3 glc-gal linkage is catalyzed by B3GAT3 [64,65].

Although likely related to other glycosylation pathways, enzymes catalyzing identical linkages have recently been linked to cancer, and are of therapeutic interest because they have been demonstrated to be epigenetically regulated. For example, *XYLT1*, *B3GALT6*, and *B3GAT3* were all found to be hypomethylated in multi-drug resistant A549 lung cancer cells compared to progenitor A549 cells [66]. Similarly, B3GALT4, closely associated with ganglioside biosynthesis, has been linked to neuroblastoma tumors in a genome-wide methylation screen [67]. *B4GALT1* has been found to be hypermethylated in invasive colorectal cancers, and was shown to be re-expressed upon treatment with the DNA methyltransferase inhibitor 5-Aza-dc [68,69]. Conversely, in breast cancer, estrogen-induced expression of B4GALT1 is associated with enhanced breast cancer cell proliferation, and thus estrogen receptor agonists have been suggested as a potential therapeutic approach [70]. These seemingly opposing roles of B4GALT1 highlight the context-dependence of HSPG regulation and function.

### 2.2. Elongation of the Tetrasaccharide Linker to Form the HS Chain: Exostosin Family

The 5-member exostosin family of genes, which consists of exostosin (EXT) and exostosin-like (EXTL) genes, is required for elongation of the tetrasaccharide core. Although there is some confusion about the possible redundancy and overlapping function of these enzymes in cell lines [71,72,73], the importance of the family is illustrated by the disparate genetic disorders which are associated with mutations in exostosin gene family members [74,75]. Evidence from in vitro experiments suggests that EXTL2 is the key enzyme for the initiation of elongation of the linker tetrasaccharide, adding the required *N*-acetyl-d-glucosamine (GlcNAc) [76] and may control HSPG biosynthesis [77,78]. EXTL3 (uniprot entry O43909) appears to have identical enzymatic activity by similarity with EXTL2 [79]. As with the EXTL2/3, EXTL1 also only adds GlcNAc residues to the growing chain. Unlike the exostosin-like (EXTL) family members, EXT1/2 catalyze the addition of both α-d-glucoronate (GlcA) and GlcNAc [80] and are therefore required for the addition of at least every other monosaccharide in the growing chain. As EXT2 does not harbor significant glycosyltransferase activity in the absence of EXT1, the EXT1/2 heterooligomeric complex localized in the Golgi is essential for the polymerization of HS [81], although recent data suggest that EXT1 deficient cells may produce shorter HS chains [82]. In vivo, mutations in *EXT1*, -*2* or -*3* lead to Multiple Hereditary Exostoses, a disease that starts with benign outgrowths termed exostoses or osteochondromas, which may develop into chondrosarcomas [75,83,84]. Interestingly, the condition may be modeled in mice and potentially treated with bone morphogenic protein [85]. However, EXT2 is also associated with seizures-scoliosis-macrocephaly syndrome without exostoses [86] while mutations in *EXTL3* are linked to skeletal abnormalities and neurodevelopmental defects with severe combined immunodeficiency in some cases [74,87,88].

EXT1 is the first of several enzymes in the HS biosynthetic pathway for which strong evidence for epigenetic regulation exists. As EXT1 plays a fundamental role in the elongation of HS chains, the epigenetic regulation of this enzyme has the capacity to affect many downstream HS functions, and impact carcinogenesis. *EXT1* is hypermethylated in leukemia (especially acute promyelotic leukemia and acute lymphoblastic leukemia) and nonmelanoma skin cancer in a screen of 454 primary tumors of different types and 79 human cancer cell lines, implying that epigenetic regulation of EXT1 is linked to oncogenesis [89]. Epigenetic silencing of *EXT1* by hypermethylation in the promoter region results in loss of HS synthesis and promotes tumor progression in cancer cells, which can be reversed by a DNA demethylating agent [89]. Recently, however, EXT1 was found to be elevated in the liver and plasma of an animal model of cholangiocarcinoma (CCA) [90]. The fact that EXT1 levels rose as early as 1 month before tumor development, and that it was also found to be elevated in the plasma of human patients with CCA, indicate that it might be useful as an early diagnostic biomarker of the disease.

### 2.3. Modification of the HS Chain: Formation of Domains

The HS chain that results from the activity of the combined exostosin protein activities is a repeating dimer of glucoronic acid and *N*-acetylglucosamine residues. This chain is then modified in a series of sequential steps and involves four separate sulfation reactions and conversion of d-glucuronic acid residues to l-iduronic acid. The combined activities of these enzymes result in a clustering of modifications into *N*-sulfated regions (NS domains) and non-sulfated regions rich in GlcNAc (NA- or *N*-acetylated domains). NS domains consist of sulfated regions containing GlcNAc, GlcA, and IdoA in contrast to the NA domains, which are non-sulfated regions composed of GlcNAc and IdoA. The activity of these enzymes is thought to be determined by cell type so that a core protein may be modified differently in a tissue-specific fashion [91,92,93]. As several of these modification enzymes are subject to epigenetic regulation, the modification of HS chains may be modulated therapeutically [92,93].

#### 2.3.1. Modification of the HS Chain: Glucosaminyl *N*-Deacetylase/*N*-Sulfotransferases (NDSTs)

This family of four enzymes with two subtypes NDST1/2 and NDST3/4 with overlapping but distinct specificities and functions is necessary for the modification of the HS chain [94,95,96]. The enzymes are bifunctional and remove an *N*-acetyl group from glucosamine (GlucNAc), replacing it with a sulfo group to form *N*-sulfated heparosan, the substrate for subsequent modifications in the HSPG biosynthetic pathway [96]. Specifically, cells not expressing *NDST1* or -2 may be 6-*O*-sulfated but not modified by 2-*O*- or 3-*O*-sulfotransferases. Overexpression or deletion of members of this family alters the composition of the HS on HSPG [97,98] and it has been suggested that some initiate HS modification/sulfation reactions, whereas others later on fill in or extend already modified HS sequences [94]. NDST2 knockout mice have defects in mast cell proteases [99,100] which can mold the tumor microenvironment. Recently, NDST4 was identified as a tumor suppressor linked to colorectal cancer [101]. Consistent with its role in colon cancer [101], mice deficient in NDST4 show altered development and homeostasis of the colonic epithelium [53] and this locus is linked to circulating resistin levels [102]. Interestingly, NDST4 has also been associated with reading disability and language impairment [103], mirroring the autosomal recessive intellectual disability linked to NDST1 [104,105] and schizophrenia and bipolar disorder linked to NDST3 [106,107]. Additional links between genes of this family and cancer may be delineated in the future as NDSTs may determine length of the HS chain [108] and are considered to be key in forming domains within the HSPG that are fundamental to determining protein interactions [95,104,105,109].

The potential tumor suppressive qualities of NDSTs [101] underscore the growing interest in modifying their expression via manipulation of their epigenetic regulation. Treatment of H-HEMC-SS chondrosarcoma cells with 5-Aza-dc decreased *NDST1* promoter methylation, increasing *NDST1* mRNA expression, and reducing their proliferative and invasive properties [110]. However, methylation-dependent regulation of *NDST1* is cell type dependent. In prostate cancer cells, *NDST1* expression was increased following 5-Aza-dc treatment in androgen-dependent non-metastatic LNCaP cells, but was unaffected in the androgen-independent metastatic PC3 cell line [93].

*NDST1* expression can also be epigenetically regulated via direct microRNA (miRNA) targeting, but the influence of miRNA-dependent regulation of *NDST1* expression on cancer prognosis and progression seems to be context dependent (e.g., affected by experimental conditions, cancer and cell type). For example, in the gastric carcinoma cell line MGC803, miR-191 targeting of *NDST1* suppresses apoptosis and promotes cancer cell growth [111]. However, in HUVEC cells, the downregulation of *NDST1* by miR-24 reduced HS chain formation and the chemotactic response to growth factor treatment [112]. Similarly, the de-repression of *NDST1* following the downregulation of its targeting miRNA, miR-149 was associated with chemoresistance and an unfavorable diagnosis in Her2-positive and basal breast cancers [113]. Interestingly, in this study, control of miR-149 expression was shown to be methylation dependent adding yet another layer to *NDST1* epigenetic control.

#### 2.3.2. Modification of the HS Chain: d-Glucuronyl C5-Epimerase (GLCE)

By changing the stereochemistry of the C5 chiral center, this step converts d-glucuronic acid residues adjacent to *N*-sulfate sugar residues in heparosan-*N*-sulfate to l-iduronic acid residues in the maturing HS chain [114]. This is important for further modifications that determine the specificity of interactions between these glycosaminoglycans and proteins as this step is required for the formation of NS-domains (*N*-sulfated disaccharide units) which are distinct from NA domains (*N*-acetylated disaccharide units) and are key for associations of proteins. There is some interplay between GLCE, exostosin, and HS2ST activity as overexpression of *GLCE* increased HS chain length but this effect was abolished by simultaneous overexpression of HS2ST and this was not seen with expression of mutant *GLCE* [115].

The dysregulation of *GLCE* expression has been observed in many cancer types, but its exact role in cancer progression is not clear. In breast and lung cancers, GLCE has a demonstrated anti-proliferative effect, but its overexpression in prostate cancer cells is associated with a much more aggressive phenotype [116,117,118]. In breast cancer cells, epigenetic regulation of *GLCE* is thought to be dependent on chromatin structure and not DNA methylation as *GLCE* expression was significantly increased following histone deacetylase (HDAC) inhibitor treatment, but was unaffected by 5-Aza-dc treatment [119]. Conversely, in prostate cancer cells, GLCE dysregulation appears to be largely mediated via aberrant *GLCE* promoter methylation, which varies dramatically between prostate cancer cell types and has been proposed to be a potential contributor to intratumor heterogeneity [120].

#### 2.3.3. Modification of the HS Chain: The *O*-Sulfotransferases HS2ST, HS6ST, and HS3ST

Heparan sulfate chains are sequentially modified by sulfation on C2, C6, and C3 by three different families of sulfotransferase enzymes (HS2ST, HS6ST, and HS3ST, respectively). As there are multiple isoforms of the HS6ST and HS3ST enzymes, a total of eleven sulfation enzymes exist which leads to variability in sulfation during HS biosynthesis. HS2ST catalyzes the sulfation on the C2 of both l-iduronyl (IdoA) and d-glucuronyl (GlcA) acid residues with a strong preference for IdoA [121]. HS2ST is required for Erk/Mapk signaling and knockout mice are perinatal lethal, potentially due to increased sulfation on other carbons, maintaining the level of HS sulfation and charge [122,123,124]. As HS3ST requires that the HS chain be previously modified by HS2ST, any compensatory sulfation must be catalyzed by HS6ST which does not require prior 2-*O*-sulfation. Thus, although 2-*O*-sulfation appears to be largely restricted to NS-domains while 6-*O*-sulfation is seen in domains with and without *N*-sulfation [125]. HS6ST may partially substitute for a lack of HS2ST activity, an activity that is key to maintaining growth factor binding sites [122,123]. In addition, as the 6-*O*-sulfations of heavily sulfated NS-domains [126,127] are targets of both SULF1 and SULF2 (see below), the binding domains for growth factors and other proteins may be edited after synthesis is complete. The family of seven HS3ST isoenzymes are thought to catalyze the rate limiting step in sulfation, producing either antithrombin III or herpes simplex binding envelope protein binding sites [128,129]. However, this site classification is undoubtedly simplistic. 3-*O*-sulfatation was recently shown to enhance Wnt binding [130] and other binding sites are likely to follow.

Although sulfation is thought to be extremely important in determining the binding of associated proteins, much remains to be determined about the importance of variations in sulfation patterns in cancer. In prostate cancer, stress-induced transcription factors increase transcription of HS2ST to increase metastatic potential [49] and the 2-*O*-sulfate modification is linked to angiogenesis [131]. Expression of the HS3ST3B1 isoform of the 3-*O*-sulfotransferase has been shown to regulate breast cancer invasiveness [132]. Interestingly, the anticoagulant heparin is much less sulfated, at 0.8 sulfates per disaccharide rather than the typical HS chain at 2.3 per disaccharide [1] but despite this, there is a large degree of overlapping specificity in protein binding between heparan and heparin [133]. Thus, much more work is needed in order to understand how this large family of sulfation enzymes functions to regulate HS structure and function.

There is currently a paucity of data regarding the epigenetic regulation of *O*-sulfotransferases, though evidence is starting to suggest its relative importance in cancer. In addition to genes involved in initiation of HS chain formation, several *O*-sulfotransferases (HS3ST1, HS3ST2, HS3STSA1, HS3ST3B1) were also found to be hypomethylated in multidrug resistant A549 lung cancer cells compared to the A549 progenitor cells [66], though the downstream effects of this hypomethylation remain uninvestigated. In H-EMC-SS chondrosarcoma cells hypermethylation, and thus decreased expression of the 3-*O*-sulfotransferases HS3ST1, HS3ST2, HS3ST3A1 was associated with a more invasive phenotype [134]. Importantly, treatment with 5-Aza-dc restored both 3-*O*-sulfotransferase gene expression and HS sulfation patterns, and reduced the proliferative and invasive capacity of the chondrosarcoma cells [110]. Similar patterns of hypermethylation of *HS3ST2* and increased tumor invasiveness have also been observed in lung and bladder cancers indicating substantial tumor suppressive properties associated with proper HS3ST2 function and HS chain sulfation [135,136].

### 2.4. Summary of Modification of the HS Chain: Complexity, Redundancy, and Protein Interactions

Several authors have proposed complex regulatory interactions between proteins involved in HS biogenesis [71,115,137,138,139]. Esko and Selleck first suggested that a macromolecular GAGosome enzyme complex [140] may function to integrate the complex regulation of the enzymes in the HS biosynthetic pathway. This proposed complex may include the family of exostosin enzymes as the length of the HS chain may be regulated by NDST, a proposed member of the GAGosome [108], and there is increasing experimental evidence to support such a complex [115,141,142]. Recently, Zhang et al. have proposed that the enzymes involved in the modification of HS chains are tightly regulated to read and write a code involved to synthesize the appropriate HS [143]. Although this is an evolving story, the regulation of HS modification enzymes is likely to be key to the overall function of HSPG.

As the exact modification of the heparan sulfate moiety is key to its function, the binding of proteins to HSPG will also be greatly affected by the exact HS structure. For example, fibroblast growth factor (FGF) 1 and 2 binding to HS chains of differing composition varies with significant implications for signaling through FGF receptor [144] and this differential growth factor signaling has been proposed to have implications for cancer [145]. Differential binding of growth factors to HS chains may affect morphogenesis [29,146,147]. In addition, HS composition affects the activity of the complement cascade which affect inflammation [148] and unsubstituted glucosamine residues inhibit heparanase activity [149] which has been implicated in metastasis. Thus, given the potent pleotropic effects of HS composition on downstream events, it is not surprising that recent work has focused modulating the activity of HS modification enzymes and on screening for HS binding affinity [150].

## 3. Heparan Sulfate Modification and Degradation Enzymes

Once synthesized, the HS chain of an HSPG may be modified or degraded by extracellular enzymes. Modification involves removal of the sulfo group at the C6 position in regions of the HS chain by SULF1/2 to reduce signaling by heparin-dependent growth factors and tumor growth [151]. In addition, the HS chain may be shortened or degraded by heparanase (HPSE1/2) to functionally remove growth factor binding sites and affect cancer progression [152].

### 3.1. SULF1 and SULF2

These extracellular enzymes remove sulfo groups from the C6 position, reducing the overall sulfation (and negative charges) of HSPGs. As SULF1/2 modify the sulfate-rich NS domains more than the glucuronic acid-rich NA domains, the domains closely associated with growth factor binding are predominately altered [137,153]. Despite functional similarities between SULF1 and SULF2, the roles of these two enzymes in the pathogenesis of cancer seem to diverge. SULF1 has been shown to exhibit tumor suppressive properties in hepatocellular carcinomas (HCC), as well as breast and ovarian cancers [154,155], potentially acting by reducing growth factor binding and signaling in expressing cells [156,157]. In contrast, SULF2 has been shown to promote growth and metastasis of solid tumors [158]. However, upregulation of both SULF1 and SULF2 has been shown to be a poor prognostic indicator for gastric cancer patients [159]. In addition, expression of SULF1 or -2 has been shown to affect response to multiple cancer chemotherapeutics. In HCC, *SULF1* induction has been shown to increase histone acetylation, and can potentiate the apoptotic effects of HDAC inhibitors [160] while *SULF2* methylation is negatively associated with cisplatin sensitivity [161]. Conversely, in lung cancer, *SULF2* methylation increases sensitivity to topoisomerase-I inhibitors by inducing *IGS15* expression, subsequently enhancing the apoptotic response [158].

The expression of both *SULF1* and *SULF2* can be regulated by the degree of promoter methylation. Hypermethylation of *SULF2* is associated with increased chemosensitivity and better survival of lung adenocarcinoma patients [158]. Contrarily, hypomethylation of *SULF1* is commonly observed in HCC, but its expression can be restored with 5-Aza-dc treatment [155]. Interestingly, while *SULF1* expression is unaffected by HDAC inhibition, restoration of *SULF1* expression by 5-Aza-dc potentiated the response of HCC to HDAC inhibitor treatment compounding the growth suppressive apoptotic affects [160]. Thus, HCC may represent a cancer type in which combinatorial epigenetic therapies could be particularly effective.

*SULF1* expression has also been demonstrated to be controlled by miRNA regulation as well. In gastric cancer patients, overexpression of miR-516-3p is associated with decreased *SULF1* expression and increased survival [162]. However, in HCC, miR-21-mediated inhibition of *SULF1* and *PTEN* promoted growth factor signaling and tumor progression [163]. Thus, as with the majority of modes of epigenetic regulation, the effects of miRNA-dependent control of *SULF1* appear to be context and cell type dependent.

### 3.2. Heparanase

Heparanase (HSPE) is an endoglycosidase that participates in the degradation and remodeling of the HS portion of HSPGs and is overexpressed in many cancer types. HSPE is associated with increased cancer metastasis, chemoresistance, and reduced patient survival. The enzyme is selective for the glycosidic bond between a glucuronic acid and an *N*-sulfo glucosamine but not a 2-*O-*sulfated iduronic acid. This action particularly affects the NS domains rich in sulfated residues and alters the binding of growth factors and other proteins [164,165]. HSPE is essentially inactive at neutral pH [166] but is activated under acidic conditions, such as tumor invasion, hypoxia, and inflammatory processes, and it activates a variety of signaling pathways [167,168,169,170,171]. As such, small molecule inhibitors of heparanase, such as Roneparstat are under intense investigation. Promising work has recently demonstrated that Roneparstat treatment during or after chemotherapy can diminish tumor regrowth in multiple myeloma patients (ClinicalTrials.gov Identifier: NCT01764880) [172,173].

Significant evidence for HPSE epigenetic regulation exists as well. *HPSE* overexpression was shown to result from promoter hypomethylation in prostate cancer [174]. In neuroblastoma cells, *HPSE* overexpression is induced by the expression of miR-558, which increased cell growth and invasive capacity [175]. However, in breast cancer cells, miR-1258 targeting of *HPSE* reduced its expression and blocked in vitro cell invasion and metastasis [176].

Though the HPSE homolog HPSE2 also binds heparan with high affinity, it lacks heparanase activity and can thus competitively inhibit HPSE function. As such, HPSE2 has been suggested to have anti-metastatic features, and its strong downregulation in infiltrating ductal adenocarcinomas has led to its proposal as a diagnostic indicator [177]. Currently, no direct evidence of *HPSE2* epigenetic regulation is available, but its expression was shown to be induced in vascular endothelial cells by homocysteine metabolites, which suggests a potential correlation between HPSE2 regulation and methyl-donor availability [178].

**Figure 2:**
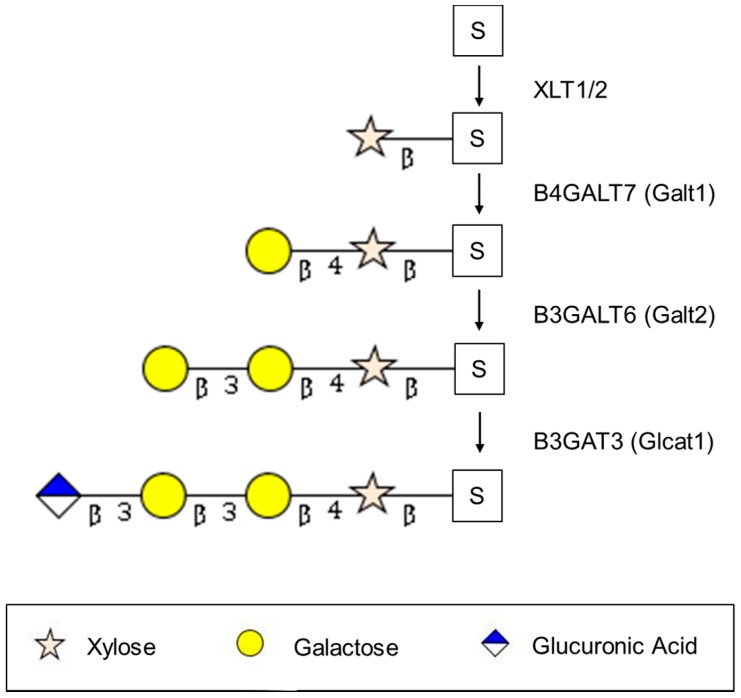
The initial step in the synthesis of the heparan sulfate proteoglycan (HSPG) linker is the linkage of xylose to a serine residue in the core protein. Subsequent linkages are catalyzed by the family sugar transferases as indicated in the text.

**Figure 3:**
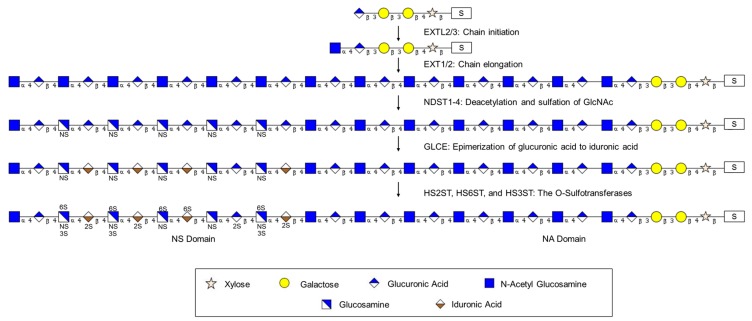
Initially, the EXT (exostosin) family of enzymes are responsible for heparan sulfate (HS) chain initiation and elongation from the serine residue (S) on a core protein. Deacetylation and sulfation of HS is performed by the NDST (*N*-deacetylase/*N*-sulfotransferase) family of enzymes. Enzymatic activity of GLCE (d-glucuronyl C5-epimerase) results in epimerization of glucuronic acid to iduronic acid on the HS chain. Finally, the HSxST family of enzymes (heparan sulfate *O*-sulfotransferases; *x* = 2, 3, or 6) catalyzes additional sulfation of the HS chain on C2, C3, and C6, respectively, within the NS domains.

**Figure 4:**
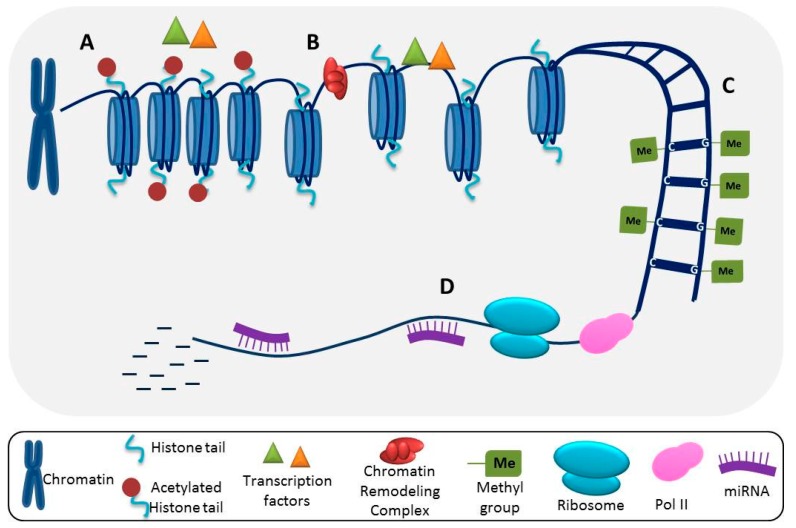
Several epigenetic mechanisms can lead to altered gene expression in tumor cells. (**A**) Histone acetylation results in tightly wound chromatin and inaccessibility of transcription factors to DNA; (**B**) Histone deacetylation and chromatin remodeling complexes promote the unwinding of chromatin complexes, allowing for transcriptional upregulation; (**C**) DNA can be methylated on C and G residues, which can lead to transcriptional repression; (**D**) Following transcription initiation by RNA polymerase II (Pol II), miRNAs can prevent protein translation by either blocking translocation of ribosomes down the mRNA transcript or by directing mRNA degradation.

Figure 2 summarizes the synthesis of the tetrasaccharide linker, while Figure 3 summarizes the elongation and modification of the HS chain. Figure 4 illustrates how gene expression may be regulated by different epigenetic mechanisms. The epigenetic regulation of these enzymes in cancer is delineated in Table 1.

## 4. Conclusions and Future Directions

Given the roles of HS signaling in oncogenesis, and interactions with the tumor microenvironment, one can begin to appreciate how components of this pathway may provide a novel set of targets for cancer diagnostics and therapeutics. The fact that many of the genes involved in HS biosynthesis are epigenetically regulated makes them particularly attractive targets, but as illustrated above, conventional epigenetic therapies, such as DNA demethylase and histone deacetylase inhibitors, may not prove particularly successful as their effects are both cancer and cell type specific. Furthermore, these types of broad-spectrum inhibitors impact far more than a given gene of interest, which can both lead to severe side effects and result in worsening outcomes [199,200].

Targeting a precise gene of interest then could improve specificity and therapeutic efficacy. However, HS biosynthesis and cancer progression are multi-faceted processes in which a number of genes can be dysregulated, often at the same time, thus a single gene candidate may not be able to be pinpointed. For example, multiple genes involved in HS biosynthesis have been shown to be differentially expressed between normal and malignant human plasma cells [186,201]. Adding to this complexity, differential transcription patterns of HS biosynthetic enzymes have been identified between cancer cells from the same type of cancer [93]. Furthermore, even targeted upregulation of a given HS biosynthetic enzyme has been demonstrated to differentially affect the expression of other HS metabolic enzymes in distinct cell types derived from the same cancer [42,64,65,93]. Another approach then might be to take advantage of the fact that these changes in transcription patterns can lead to specific glycosignatures that can then be recognized and targeted.

Ultimately, the end products of these enzymatic processes are what are impacting the cellular events that eventually lead to malignancy. For example, changes in HS chain sulfation have been linked to both the progression of cancer and cancer invasiveness [202]. Furthermore, protein glycosylation has been demonstrated to play a role in every recognized cancer hallmark, so our ability to interpret the glycocode will be crucial to the development of improved cancer therapies [203]. Indeed, some of the most common clinically used biomarkers for cancer diagnosis and monitoring are glycoproteins, and are known to be aberrantly glycosylated in cancer (e.g., PSA for prostate cancer; MUC16 for ovarian cancer; and SLe^a^ for pancreatic cancer) [204]. However, current assays assessing these proteins lack the necessary specificity and sensitivity for early cancer detection, thus the identification of specific glycoforms of a certain protein could improve diagnostic potential and, theoretically, improve patient prognosis.

One approach to begin deciphering the cancer glycome is using high-throughput glycan and lectin arrays for the identification of glycan substrate specificities and changes in glycan moieties. As lectins can distinguish between linkages and nuances of glycan moieties, they can serve as very sensitive and precise detectors of changes in glycoprotein structures. Recently, glycan array analysis and lectin profiling identified a novel lectin overexpressed in pancreatic cancer and was able to detect pancreatic cancer with higher sensitivity than the current best biomarker, SLe^a^ [205]. Further, in a subset of breast cancer patients, lectin-binding profiles were able to distinguish glycan binding differences within the serum from metastatic and non-metastatic patients [206]. Lectin arrays have also been used to identify key changes in glycosylation patters for the monitoring of cancer cell metastasis and response to treatment [207,208]. These findings illustrate the utility of lectin-based arrays for both clinical and research applications in cancer.

Lectin arrays can also been used to isolate and characterize the glycosignatures of extracellular vesicles [209,210]. Extracellular vesicles play a key role in intercellular communication by transporting biomolecules from cell to cell, and are released by virtually all cell types, yet seem to play a particularly vital role in the transmission of pathogenic signaling disease processes such as cancer. Intriguingly, extracellular vesicles are rich in glycoconjugates, and cancer cell-derived extracellular vesicles exhibit unique glycan profiles which depend on cell surface HSPGs for their internalization and functional activity [39,211]. Thus it is exciting to think of the potential to utilize glycan- and lectin-based arrays to identify cancer these cancer-specific glycosignatures to manipulate extracellular vesicle uptake and delivery based on the unique HSPG network of a given cancer cell type.

In this review, we have detailed an argument that epigenetic based therapies focused on the HSPG biosynthetic pathway have tremendous potential for the treatment of multiple types of cancers. The importance of HSPGs in oncogenesis is demonstrated by the fact that potential biomarkers exist for the progression of many disparate cancers. Epigenetic therapies are likely to act on multiple levels as HS structure affects growth factor signaling and sensitivity to chemotherapeutics. The importance of changes in HS composition supports utilization of lectin-based arrays to guide the development of these therapies.

## Figures and Tables

**Table 1:** Heparan sulfate proteoglycan (HSPG) enzymes involved in tumor progression.

Enzyme	Major Function	Expression Change	Possible Therapeutic Targeting	Type(s) of Cancer	References
XYLT1/2	Addition of xylose to a serine on a core HSPG to initiate HS chain synthesis	Up	shRNA targeting of XYLT1; DNA methylating agents	Breast cancer/bone metastasis; breast cancer associate fibroblasts; multidrug resistance	[66,179,180]
B4GALT1	Formation of the β 4 xyl-gal linkage	Varied	5-Aza-dC treatment; estrogen receptor blockers	Colon cancer; breast cancer	[68,69,70,181,182,183]
B3GALT6	Formation of the β 3 gal-gal linkage	Up	Liver X receptor agonists	Colon cancer	[184]
B3GAT3	Catalyzes the β 3 glc-gal linkage	Up	DNA methylating agent	Multidrug resistance	[66]
EXT1/2	Catalyzes the addition of both α-d-glucoronate (GlcA) and GlcNAc during HS chain elongation	Varied	5-Aza-dc treatment	Osteochondromas, cholangiocarcinoma, leukemia	[89,90]
EXTL1/2/3	Adds the required *N*-acetyl-d-Glucosamine (GlcNAc) for elongation of the HS chain	Down	5-Aza-dc treatment; siRNA	Colon cancer	[185]
NDST1-4	Replaces the *N*-acetyl groups (GlcNAc) with *N*-sulfate groups (GlcNS) on a glucosamine residue	Varied	5-Aza-dc treatment; miRNA interference	Colon cancer (NDST4); breast cancer	[101,112,113,177]
GLCE	Converts glucuronic acid (GlcA) to its epimer iduronic acid	Varied	Cancer-type dependent; ectopic overexpression improves breast and lung cancer prognosis, while overexpression is associated with increased aggressiveness in prostate cancer	Breast cancer; lung cancer; prostate cancer	[93,116,117,118,119,120]
HS2ST1	Mediates 2-*O*-sulfation of both l-iduronyl and d-glucuronyl residues within the maturing HS	Up	Heparin treatment; histone methyltransferase inhibitor	Breast cancer; multiple myeloma	[186,187,188]
HS6ST1-3	Catalyzes the transfer of sulfate from 3-Phosphoadenosine 5-Phosphosulfate (PAPS) to position 6 of the *N*-sulfoglucosamine residue (GlcNS) of heparan sulfate	Up	HS6ST inhibitors and HS mimetics	Ovarian cancer; breast cancer; pancreatic cancer	[59,189,190,191]
HS3ST1-6	Utilizes 3-phospho-5-adenylyl sulfate (PAPS) to catalyze the transfer of a sulfo group to position 3 of glucosamine residues in heparan	Down	5-Aza-dc treatment	Breast cancer; invasive ductal carcinomas; chondrosarcoma	[59,110,134,135,136,177,192]
SULF1	Selectively removes 6-*O*-sulfate groups from HS chains	Varied	HS mimetic (PI-88); 5-Aza-dc treatment; miRNA interference	Multiple cancers	[193,194,195]
SULF2	Selectively remove 6-*O*-sulfate groups from heparan sulfate	Up	Sulf inhibitors (OKN-007); proteasome inhibitors (bortezomib); HS mimetic (PI-88)	Multiple cancers	[154,193,194]
HPSE	Cleaves heparan sulfate proteoglycans to permit cell movement through remodeling of the extracellular matrix	Up	Roneparstat; miRNA interference; estrogen receptor antagonists	Multiple myeloma; brain cancer; breast cancer; colon cancer	[173,174,175,176,196,197]
HPSE2	Binds heparin and heparan sulfate with high affinity, but lacks heparanase activity	Down	Prognostic biomarker as elevated HPSE2 is correlated to improved outcomes	Breast cancers; head and neck cancers	[177,198]

## Abbreviations

BxGALT	β 1-x galactosyltransferase
BxGAT	β 1-x glucuronyltransferase
EXT	Exostosin
EXTL	Exostosin-like
GLCE	d-glucuronyl C5-epimerase
GPC	Glypican
HS	Heparan sulfate
HSPE	Heparanase
HSPG	Heparan sulfate proteoglycan
HSxST	Heparan sulfate x-*O*-sulfotransferase
NA domain	*N*-acetylated disaccharide units
NS domain	*N*-sulfated disaccharide units
NDST	*N*-deacetylase/*N*-sulfotransferases
SULF	Sulfatase
XYLT	Xylosyltransferase
